# A Review of Diatom Lipid Droplets

**DOI:** 10.3390/biology9020038

**Published:** 2020-02-21

**Authors:** Ben Leyland, Sammy Boussiba, Inna Khozin-Goldberg

**Affiliations:** Microalgal Biotechnology Laboratory, The French Associates Institute for Agriculture and Biotechnology of Drylands, The J. Blaustein Institutes for Desert Research, Ben-Gurion University of the Negev, Sede Boqer Campus, Midreshet Ben-Gurion 8499000, Israel; leyland@post.bgu.ac.il (B.L.); sammy@bgu.ac.il (S.B.)

**Keywords:** diatoms, lipid droplets, triacylglycerols

## Abstract

The dynamic nutrient availability and photon flux density of diatom habitats necessitate buffering capabilities in order to maintain metabolic homeostasis. This is accomplished by the biosynthesis and turnover of storage lipids, which are sequestered in lipid droplets (LDs). LDs are an organelle conserved among eukaryotes, composed of a neutral lipid core surrounded by a polar lipid monolayer. LDs shield the intracellular environment from the accumulation of hydrophobic compounds and function as a carbon and electron sink. These functions are implemented by interconnections with other intracellular systems, including photosynthesis and autophagy. Since diatom lipid production may be a promising objective for biotechnological exploitation, a deeper understanding of LDs may offer targets for metabolic engineering. In this review, we provide an overview of diatom LD biology and biotechnological potential.

## 1. Introduction

LDs are an organelle composed of a core of neutral lipids, mostly triacylglycerol (TAG), surrounded by a polar lipid monolayer [1,2]. LDs can store reserves of energy, membrane components, carbon skeletons, carotenoids and proteins [3,4]. Many different synonyms have been used to describe this organelle throughout the literature and they can vary between organisms, such as lipid bodies, lipid particles, oil bodies, oil globules, cytoplasmic inclusions, oleosomes and adiposomes. We will use the term “lipid droplet” in this review, abbreviation “LD,” due to its current wider use within the scientific community. LDs can mitigate stress caused by excess lipid, carbon or protein aggregate accumulation, serve as an energy sink during periods of electron flow imbalance or nutrient scarcity and help maintain redox homeostasis [5,6]. In so doing, they buffer the internal stoichiometry of cells from changes in their environment. Their functions are coordinated with ERAD, proteasomal degradation, autophagy, beta-oxidation, photosynthesis and lipid metabolism [7]. They serve as a metabolic network hub for the storage and exchange of proteins and lipids between multiple cellular compartments. Such inter-compartmental connections can exist as direct contact sites, close physical associations or involve vesicle trafficking [8,9]. The myriad functions of LDs are performed and regulated by a variety of proteins, which are localized to LDs by several mechanisms [10]. Although diatom lipid metabolism has been studied from a physiological perspective for several decades, recent advances in diatom molecular biology and LD biology in other organisms, are contributing to a more sophisticated and comprehensive understanding. Only a handful of diatom LD proteins have so far been characterized, although recent proteomic screens have identified further candidates for future study. In this review we will summarize LD biology within the context of diatom cellular ultrastructure, physiology, biochemistry, ecology and evolution. Although this topic was reviewed recently [11], we will describe recent research that has advanced our understanding of diatom LDs. Although this sub-discipline is in its infancy, since LDs are an ancient organelle conserved among all eukaryotes, we will draw from comparisons with other organisms to fill in the gaps. Understanding this organelle and its protein targeting mechanisms may hold the key to the biotechnological exploitation of diatoms.

## 2. Evolutionary Context

LDs are probably an evolutionarily ancient organelle that was present in the Last Eukaryotic Common Ancestor (LECA). This can be deduced by several lines of reasoning. LDs are ubiquitous among the *Eukaryota* and are even present in some bacteria. In *Acinetobacter* and *Rhodococcus*, LDs form in response to a high carbon:nitrogen ratio and by budding-off from the cytoplasmic membrane [12]. This is in contrast to eukaryotes, where the most commonly accepted models suggest they bud from the ER. Protein and lipid trafficking to LDs has been shown to involve proteins such as coatomers, Arf1, SNARE, TRAPP and Rab GTPases [13,14,15,16,17]. This conserved machinery was present in the LECA and was probably responsible for the differentiation of the endomembrane system into specialized compartments [18,19].

**Figure 1:**
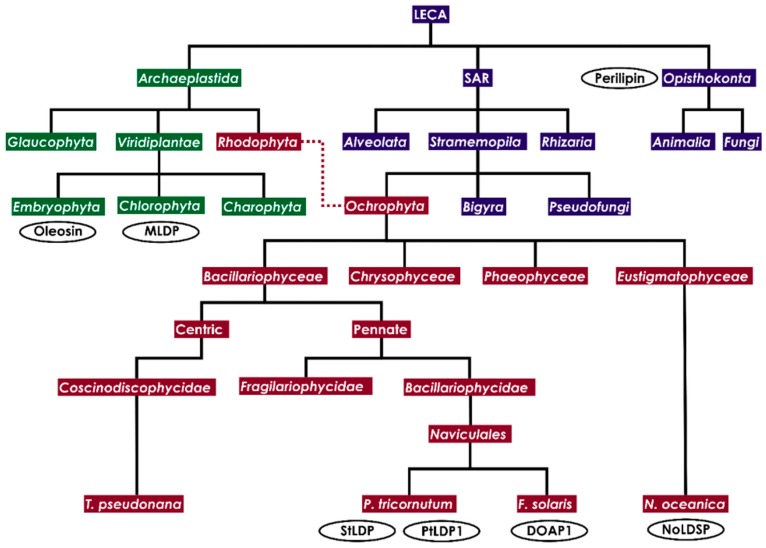
Cladogram illustrating major eukaryotic lineages in relation to diatoms. Thick black lines indicate nuclear inheritance; dotted line indicates plastid inheritance (intermediate stages not shown). Encircled in black font are representative lipid droplet proteins unique to model lineages. LECA = Last Eukaryotic Common Ancestor, MLDP = Major Lipid Droplet Protein, StLDP = Stramenopile Lipid Droplet Protein, PtLDP1 = *P. tricornutum* Lipid Droplet Protein 1, DOAP1 = Diatom Oleosome Associated Protein 1, NoLDSP = *N. oceanica* Lipid Droplet Surface Protein.

At some point over a billion years ago, the ancestors of the *Opisthokonta*, *Archaeplastida* and SAR clades diverged from the LECA [20]. The SAR super group contains the *Rhizaria*, *Alveolata* and *Stramenopila*; the *Opisthokonta* contain animals and fungi; and the *Archaeplastida* contain the *Rhodophyta* (red algae) and *Viridiplantae* (green algae and plants) [21]. Diatoms (*Bacillariophyceae*) are members of the *Ochrophyta*, a monophyletic group within the kingdom *Stramenopila*, which gained a complex red plastid by a serial endosymbiotic process [22,23] (Figure 1). Most research on LDs has been performed on model animal species and cell lines, yeast, green algae and plants. Although there has been a plethora of recent advances on the study of diatom LDs and lipid metabolism in recent years, potential explanations to “fill in the gaps” can be acquired by examination of other model organisms. Much research on diatoms infers similarities with other algae, simply by virtue of being algae and thus sharing similar physiological, ecological and plastidial characteristics. However, the algae are a polyphyletic group and in fact diatoms share some things more in common with animals than the *Viridiplantae*, such as an ornithine-urea cycle [24]. Considering the antiquity of LDs, we can therefore anticipate that diatoms may equally share features in common with the *Opisthokonta* as with the *Viridiplantae*. When possible, comparisons with other stramenopiles can be given preference, such as with the well-studied eustigmatophyte *Nannochloropsis*.

The fossil record and molecular clocks suggest that diatoms likely originated and proliferated during the Mesozoic era [25,26,27]. During the Cenozoic, geologic shifts, such as the opening of the Drake passage and subsequent formation of the Antarctic Circumpolar Current, resulted in seasonal nutrient pulses which allowed for the domination of marine phytoplankton communities by diatoms [28]. Antarctic sea ice diatoms rely on TAG as an energy sink to cope with the stressful conditions of their environment [29]. The success of diatoms in-turn drove the evolution of krill, cetaceans and other components of the rich marine food-webs that characterize contemporary oceans [28]. Therefore, understanding LDs and diatom lipid metabolism is not merely an esoteric exercise but one of several explanatory variables within a broader ecological and evolutionary context.

## 3. Lipid Composition

### 3.1. The Core

The LD core is composed of neutral storage lipids. Although such lipids are usually in the form of triacylglycerol (TAG), the LD core in some organisms and cell types can consist, in part or entirely, of sterols, other steryl esters, wax esters, carotenoids or polyprenols [4,12,30,31]. Nevertheless, TAGs are the primary storage lipid in diatoms [32,33]. TAGs are an inert, stable, hydrophobic molecule consisting of three fatty acids esterified to a glycerol backbone. TAGs store large amounts of energy, carbon and membrane components, while simultaneously shielding the cell from the potential cytotoxicity of free fatty acids [34,35,36]. Thin-layer chromatography of LDs isolated from *P. tricornutum* confirms they are indeed composed mostly of TAG [37]; however recent lipidomic analysis has suggested that some sterols are present in lower abundance [38].

Various species of green algae sequester carotenoids in LDs, which may shield the photosynthetic apparatus from excessive light, reduce reactive oxygen species, serve as a sink for excess photosynthates or protect LD lipids from oxidation. Evidence of several pigments in *P. tricornutum* and *F. solaris* LDs has been found, including beta-carotene and fucoxanthin, as well as fucoxanthin-chlorophyll a/c binding proteins [37,38,39]. Our lab has made similar observations (unpublished) but the presence and function of carotenoids or other isoprenoids, in diatoms has not been explored in detail yet.

LDs can be labeled with neutral lipid-specific fluorochromes, such as Nile Red, Bodipy, LD540 or LipidTOX, which can aid in their visualization and quantification in vivo [40,41,42,43]. Developments in Raman spectroscopy are also yielding promise for label-free in vivo measurements of LDs, including the potential to differentiate different lipids [44,45,46]. The low buoyancy of LD neutral lipid content allows for their isolation by density gradient centrifugation, since they float on the surface of the aqueous phase of centrifuged lysate [47,48].

### 3.2. The Monolayer Membrane

Most organelles are bounded by a polar lipid bilayer; however, LDs are unique in that they are bounded by a monolayer. This is not a coincidental phenomenon but a more thermodynamically stable configuration. The phospholipids constituting standard bilayers have a hydrophilic head and a hydrophobic tail, whereby the bilayer is formed by the sandwiching together of the two hydrophobic tails of each adjacent layer, such that the organelle contains an aqueous core. However, since LDs have a hydrophobic core, they no longer require the inner phospholipid layer of the bilayer and instead are bounded by a monolayer in which the hydrophobic tails are pointed inwards towards the core. In most eukaryotes, the phospholipid monolayer of LDs is typically composed of phosphatidylcholine (PC) and phosphatidylethanolamine (PE) [49], however in microalgae other lipid classes have been observed, including chloroplast glycerolipids and betaine lipids [50,51]. Lipidomic analysis of nitrogen starved *P. tricornutum* (Pt.1) identified the major polar lipids to be sulfoquinovosyldiacylglycerol (SQDG), PC and the betaine lipid diacylglycerylhydroxymethyltrimethyl-β-alanine (DGTA) [38]. The polar lipid composition of the LD membrane may affect which proteins bind to it [52]. During phosphorous deficiency, phospholipids are replaced by non-phosphorous lipids, such as betaine lipids, sulfolipids and galactolipids [53,54], although it is currently unknown what effect this might have on the LD membrane.

### 3.3. Intracellular Connections

**Figure 2:**
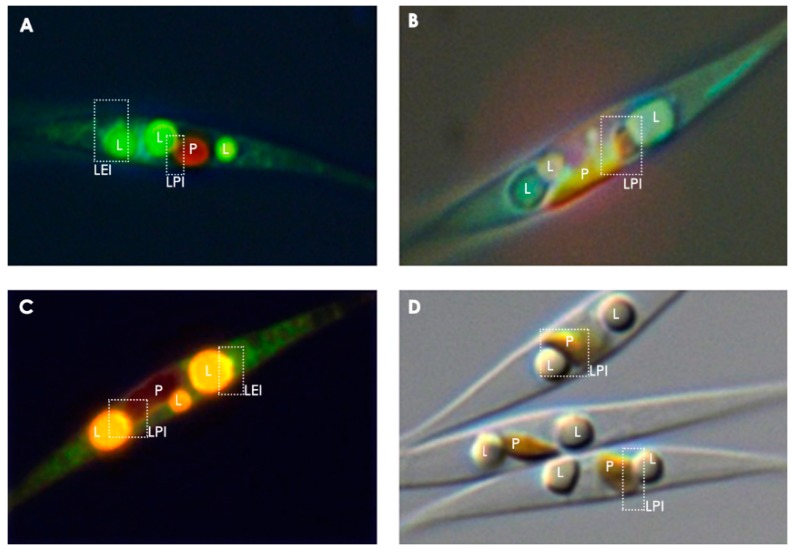
Micrographs of nitrogen starved *P. tricornutum* cells, illustrating potential interconnectivity between LDs and various cellular compartments. Plastidial autofluorescence appears red, the LD stain Nile Red fluoresces yellow and the ER/mitochondrial/endomembrane stain DiOC_6_ fluoresces green/greenish blue. (**A**) Epifluorescent image of cells stained with Nile Red and DiOC_6_, (**B**) Epifluorescent image of cells stained with only DiOC_6_, (**C**) Epifluorescent image of cells stained with Nile Red and DiOC_6_, (**D**) Differential interference contrast image with no epifluorescent staining. P = plastid, L = lipid droplet, LPI = lipid droplet-plastid interface, LEI = lipid droplet-endomembrane interface. The interfacial regions, emphasized in boxes with dotted lines, are speculated to be potential regions of interaction between LDs and other organelles.

**Figure 3:**
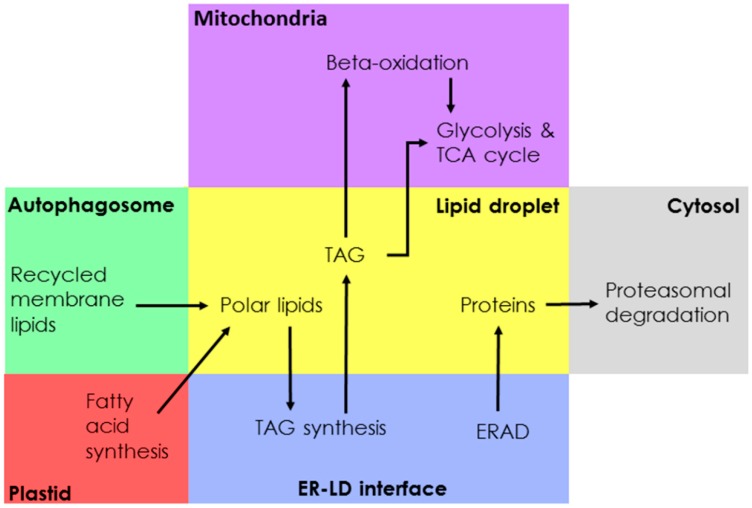
Simplified schematic of diatom LD metabolic network.

In most eukaryotes, LDs have been shown to be closely associated with the ER, which is the presumed site of LD biogenesis. In yeast, most LD proteins co-localize to the ER [7]. Direct contact via membrane bridges connecting LDs to the ER allows for the exchange of functional enzymes [55,56]. ER-Golgi transport machinery, such as coatomers and Arf1, may also be involved in the delivery of functional enzymes to LDs [13,57]. In both the *Opisthokonta* and *Viridiplantae*, seipin localizes to ER-LD contact sites and is important for LD biogenesis and maturation [58,59,60]. The *P. tricornutum* genome contains at least one seipin homologue, PHATRDRAFT_47296 (B7G3W8) [61]. Overexpression of PtSeipin resulted in increased TAG content, larger LDs, a higher proportion of saturated FAs compared to total FA and a lower proportion of unsaturated FAs [61]. In the model green alga *Chlamydomonas reinhardtii*, LDs have been shown to connect to both the ER and the plastid, the latter contributing DAG to the expanding TAG pool, as well as polar lipids and proteins [51,62,63]. In both the *Opisthokonta* and *Viridiplantae*, LDs can also form close-associations with mitochondria or peroxisomes, where they function as a conduit for FAs directed to mitochondrial or peroxisomal beta-oxidation [64,65,66,67,68,69,70,71]. In *Arabidopsis*, the peroxisome-localized TAG lipase, Sugar-Dependent 1 (SDP1), was shown to translocate to LDs during early seedling growth [72]. The interconnectivity between LDs and other cellular compartments may be more complex in diatoms compared to the *Opisthokonta* and *Viridiplantae*. The *Ochrophyta* inherited their plastid by a serial endosymbiotic process [22,23,73], resulting in continuity between the outer membrane of the diatom plastid and the nuclear and ER membranes [74,75], as well as other complex inter-compartmental interactions. For instance, metabolic cross-talk between the plastid and mitochondria, allows for the regulation of cellular redox balance and resource allocation under variable environmental conditions [76,77,78]. Potential interactions between LDs and other intracellular compartments can be seen in Figure 2 and a simplified summary of the putative interactions between LDs and other compartments is illustrated in Figure 3.

## 4. LD Formation and Degradation

### 4.1. Biogenesis

Diatoms experience fluctuations in nutrient availability and photon flux density. Being single celled organisms, they are particularly vulnerable to such dynamic conditions, in terms of maintaining metabolic homeostasis. This necessitates the ability to buffer against external changes to the environment with internal storage capabilities. LDs are one aspect of this buffering requirement and hence form in response to stress conditions, as well as part of the natural diurnal cycle. For instance, in *P. tricornutum*, storage lipid accumulation reaches its diurnal zenith at dusk and is consumed throughout the night, reaching a nadir at dawn [78,79]. Various nutrient deficiencies result in TAG accumulation, including silicon [80,81], phosphorous [82,83] and nitrogen [33,84,85,86]. Each nutrient stress condition results in different metabolic changes and hence, differences in lipid class profile, positional isomers and enantiomers [87]. The waning of the diurnal cycle and macronutrient deficiency have been shown to cause co-ordinated changes in transcriptional regulation, re-organization of metabolic flux and the reallocation of cellular carbon towards lipid production [83,85,88,89,90,91]. Similar effects can be achieved by disrupting nutrient assimilation, such as knocking-out or knocking-down nitrate reductase [92,93]. Neutral lipid accumulation can also be induced by exogenous addition of a variety of compounds, including fatty acids [94], sodium bicarbonate [95], nitric oxide [96] or other compounds [97,98]. The various stresses described above cause imbalances in energy and redox homeostasis. For example, nitrogen starvation constrains the formation of nitrogenous compounds, such as proteins, whose constant turnover is required for the operation of photosynthetic machinery [99,100,101]. Likewise, phosphorous starvation constrains the formation of ATP. Both de novo synthesis and remobilization of membrane lipids, contribute to the accumulation of storage lipids [83,102,103,104]. LDs likely form in predefined microdomains of the ER, where a lens of neutral lipids accumulates between the two leaflets of the ER membrane [105,106]. The expanding neutral lipid accretion disk leads to increased curvature of the ER membrane, which can be sensed by proteins that are further recruited to the nucleation site and contribute to LD expansion [56,107,108,109].

### 4.2. TAG Biosynthesis

Acyl groups exported from the plastid are eventually incorporated into TAG via either acyl-CoA dependent or independent pathways [110,111,112]. The acyl-CoA dependent pathway involves the sequential activity of glycerol-3-phosphate acyltransferase (GPAT), lysophosphatidic acid acyltransferase (LPAAT) and diacylglycerol acyltransferases (DGAT). In the penultimate step, phosphatidic acid is converted to diacylglycerol (DAG), which is finally converted to TAG by one of several DGATs. Many of these acyltransferases were cloned and overexpressed in *P. tricornutum* or *T. pseudonana*, where they were reported to enhance TAG generation [113,114,115,116,117,118,119]. The acyl-CoA independent pathway involves phospholipid:diacylglycerol acyltransferase (PDAT), which uses other polar lipids as an acyl donor for the formation of TAG from DAG. PDAT typically uses PC in yeast and plants [120] but can use a range of various glycerolipids in microalgae [121]. TAG biosynthesis canonically occurs in the ER, although in other eukaryotes, TAG biosynthesis enzymes have been shown to re-localize to LDs [55,56,122]. Thus, the total remobilization of intracellular constituents towards storage lipid production requires co-ordination between multiple organelles. Since LDs are at the heart of this process and can potentially physically associate with all the organelles involved, it might be possible that they function as a metabolic network hub, facilitating the interchange of the various enzymes and substrates involved. Identification of mitochondrial, plastidial and ER proteins in LD proteomics experiments might be indication of this hypothesized connection.

### 4.3. Lipolysis

During recovery from stress or during the dark period of the diurnal cycle, LD lipid stores are remobilized and used for energy, membrane components, carbon skeletons or other metabolic requirements. LD lipid stores are remobilized by two main mechanisms: either by lipolysis or by autophagic degradation. During lipolysis, TAGs are hydrolyzed, liberating their three fatty acids and glycerol for consumption by beta-oxidation and glycolysis in the mitochondria [123]. Lipolysis is performed by a variety of different lipases and other lipolytic enzymes, which may be differentially expressed under different nutrient availabilities [124,125]. In other eukaryotes, some TAG lipases and their cofactors have been demonstrated to localize to LDs [126,127,128,129]. For example, CGI-58 is a conserved protein with LPAAT activity that co-localizes to LDs with Adipose Triglyceride Lipase (ATGL), assisting in the breakdown of TAG [130,131]. A homologue of CGI-58 was identified in *T. pseudonana*, Thaps3_264297, which also displays LPAAT, phospholipase and lipase activity [124]. Knockdown of Thaps3_264297 resulted in increased TAG accumulation in a variety of conditions, including stationary phase and silicon starvation, faster TAG accumulation, larger LDs and potentially diminished membrane re-modeling [124]. *P. tricornutum* possesses a TAG lipase, Tgl1, which shares homology with a LD-localized TAG lipase in plants, SDP1 [125,132]. Knockdown of Tgl1 resulted in increased TAG accumulation during growth phase, most prominently during stationary phase. More lipases have been predicted *in silico*. The direct localization to LDs of either PtTgl1 or Thaps3_264297 has yet to be confirmed.

### 4.4. Autophagy

Autophagy is a major cellular self-degradation process which can involve the degradation of either bulk or select constituents [133]. Core autophagic proteins are conserved among most eukaryotes, including the *Stramenopila* [134]. The relationship between autophagy and LDs can be complex [135]. LDs can be digested by selective autophagy but autophagy can also contribute recycled cellular components to LD expansion [64]. For example, in starved mammalian cell lines, fatty acids liberated by autophagic degradation of membranes, were shown to be directed to LDs by DGAT1 [136]. The LDs were subsequently degraded by lipolysis, channeling fatty acids to closely associated mitochondria, while simultaneously shielding them from lipotoxicity and acylcarnitine accumulation. Furthermore, LDs can contribute membrane components for autophagosomal biogenesis [137]. Lipophagy, the engulfment of a LD by an autophagosome, is a type of selective autophagy which was shown to be coupled with both biogenesis and degradation of LDs in the *Opisthokonta* [138,139] and *Viridiplantae* [140,141,142].

In the *Stramenopila*, microlipophagy-like engulfment of LDs into vacuoles was observed in *Nannochloropsis oceanica*, the marine oleaginous eustigmatophyte [143]. A biomolecular fluorescence complementation (BiFC) assay indicated interaction between the major LD surface protein (LDSP) and the hallmark autophagy protein, AUTOPHAGY-RELATED8 (ATG8) [143]. Deletion of a predicted ATG8-interacting, conserved WxxI LIR motif in NoLDSP disrupted its association with ATG8 [143]. The major lipid droplet protein of *P. tricornutum*, StLDP displays a similar hydrophobicity pattern to NoLDSP, though they differ in amino acid sequence [37]. We also identified the ATG8-interacting motif in StLDP using the iLIR Atg8 binding motif prediction tool [144], suggesting that it may play a similar function as LDSP in *N. oceanica*.

In nitrogen-limited *P. tricornutum*, the recycling of internal nitrogenous compounds involves the upregulation of autophagosomal, proteasomal and lysosomal machinery [145]. Nonoyama et al. (2019) suggested that autophagy may be involved in LD degradation in *F. solaris* and *P. tricornutum* based on the presence of vesicle trafficking, vesicle coat and heat shock proteins in the LD proteome [39]. Administration of the inhibitor chloroquine, which impairs the fusion of autophagosomes with lysosomes [146], suppressed degradation of mature LDs [39]. Clathrin was identified in the LD proteomes of both *F. solaris* and *P. tricornutum* [38,39,147]. During recovery from nutrient starvation, LDs shrink in size due to lipolysis or lipophagy. However, introduction of the clathrin inhibitor Pitstop 2 resulted in larger LDs compared to control treatments, implicating vesicle trafficking machinery in the remobilization of LD storage lipids [39]. A similar observation was made in animal hepatocytes, where the RNA silencing of clathrin inhibited the autophagic degradation of LDs [148]. In eukaryotes, autophagy and other catabolic processes are inhibited by target of rapamycin (TOR) [149]. Under nutrient replete conditions, inhibition of TOR resulted in TAG accumulation in *P. tricornutum* [98]. Further experiments will be needed to decipher the precise relationship of autophagy with LDs in diatoms.

## 5. LD Proteins

### 5.1. The Challenges of Identifying LD Proteins

Most organelles function due to the co-ordinated operations of hundreds of different proteins, their proteome. The decipherment of the LD proteome is obfuscated by several factors. Firstly, the proteome will differ between species, strains, cell types, growth conditions and so forth. Second, many LD proteins can have multiple locations within the cell [150] or may translocate from other locations. For example, Diatom Oleosome-Associated Protein 1 (DOAP1) is translocated from the ER to LDs in *Fistulifera solaris* [151]. Sub-cellular localization can sometimes be inferred using in silico sequence-based predictive algorithm, such as HECTAR [152]. However, such predictions cannot be relied upon as empirical verification and may not always be accurate, particularly when predicting the localization of proteins to a compartment it was not programmed to take into account. To our knowledge, no such algorithm has been designed to predict LD protein localization yet. Many studies which demonstrate LD localization depend on fluorophore-tagging, such as green fluorescent protein (GFP). This is limited by the resolution of optical microscopy, which can have difficulty resolving differences between a protein physically associated with the LD or merely localized near it on an adjacent or engulfing membrane [57]. Alternative methods, such as immunogold labelling, allow for finer resolution imagery via electron microscopy, although fixation methods can occasionally result in artefacts. Proteins from isolated LDs can be identified en *masse* by liquid chromatography coupled to mass spectrometry (LC/MS). In this procedure, the proteins are typically separated on an electrophoretic gel, stained, excised and digested *in gel* with the serine protease trypsin [153]. This procedure has several limitations, including poor peptide recovery, modification artefacts and relies on effective visualization of stained protein bands. Furthermore, the high lipid content of LD samples and hydrophobic properties of some LD proteins, may interfere with standard SDS-PAGE [47]. New technological developments, such as suspension trapping, can potentially overcome such obstacles [154,155]. However, LDs can be contaminated with proteins from other cellular compartments during the isolation procedure. Moreover, since the LD membrane forms direct contact sites with other cellular membranes and may non-specifically bind hydrophobic or amphipathic proteins [156], it may be fundamentally impossible isolate a “pure” LD. A newly developed tool, APEX2, can be used both as a tag for protein imaging by electron microscopy, as well as a label for organelle-specific proteomics, thus resolving many of the difficulties to discussed above [157,158]. Nevertheless, such challenges emphasize the importance of ancillary experiments to confirm protein localization.

### 5.2. LD Protein Targeting

LDs differ from mitochondria, plastids and the ER, which require cleavable signal peptides for protein targeting [159,160,161]. So far, no such consensus signal has been identified for LD-specific protein targeting. However, multiple mechanisms have been implicated in targeting proteins to LDs and which may function in concert with each other [162]. A heterologously expressed, GFP-tagged LD protein from the green alga *Haematococcus pluvialis*, was demonstrated to localize to LDs in the diatom *P. tricornutum* [94]. This suggests a conserved mechanism of LD protein targeting shared across evolutionarily disparate kingdoms.

The amphipathic alpha helix is a structural feature of many LD proteins critical for their binding to the LD membrane [163,164,165]. Molecular dynamic simulations and in vitro experiments suggest that the outer polar lipid monolayer of LDs contains membrane packing defects that expose the hydrophobic core, facilitating the binding of amphipathic helices containing large hydrophobic residues [10,156,166]. Such alpha-helices are a physicochemical property of proteins that can vary in size and specific amino acid composition [167]. This means that LD proteins in diverse kingdoms may possess them without sharing sequence homology. Amphipathic alpha-helices can sense membrane curvature and even specific lipids, which may be factors affecting their targeting specificity [167]. Several eukaryotic LD proteins also possess hydrophobic domains that anchor them to LDs [168,169,170].

Although the general physicochemical properties of amino acids, such as size and hydrophobicity, can contribute to LD protein targeting, specific amino acid residues can be important as well. Proline residues are critical for the architecture of proline-knot motifs, hydrophobic anchors which bind some proteins to the LD membrane, such as oleosin and caleosin in plants [171,172]. Yoneda et al. (2016) [37] suggest this may be a feature of some diatom LD proteins as well. Tryptophan residues have also been demonstrated to facilitate the interfacial binding between proteins and LD membranes [162,173,174]. Other residues, such as cysteines, can be modified by post-translational modifications that facilitate membrane binding [175], discussed further below.

Multiple kinds of post-translational modifications have been implicated in LD protein targeting and regulation. For example, phosphorylation of the animal LD protein perilipin A is required for the translocation of hormone-sensitive lipase to LDs, thus increasing lipolysis [176,177,178]. Furthermore, some LD proteins may be modified with hydrophobic prenyl or acyl moieties, which facilitate their association with the LD membrane [179]. Acyl modifications are reversible, which could provide a mechanistic explanation as to how some proteins may re-localize to LDs from other locations in response to stress conditions. In mice, prenylation of ALDH3B2 is required for LD localization, where it probably detoxifies aldehydes produced by lipid metabolism [180]. Interestingly, post-translational modifications do not necessarily function in isolation but may confer targeting specificity. For example, in animal cells, ELMOD2 can localize to the ER or mitochondria but when it is modified with a palmitoyl moiety–palmitoylation–it localizes to LDs, where it activates Arf1, which subsequently recruits ATGL [181]. In cultured mammalian cells, a component of the SNARE membrane fusion machinery, SNAP23, was also shown to require palmitoylation to localize to LDs [182]. Multiple Rab GTPases are consistently identified in LD proteomic screens in various eukaryotes and several have been demonstrated to localize to LDs, where they are hypothesized to regulate interactions with other organelles [15,16,183,184]. Interestingly, prenylation of Rab proteins is required for their targeting to specific membranes [185,186]. Ubiquitination may also play a role in LD protein localization. For instance, in both plants and animals, a ubiquitin regulatory protein recruits Cdc48 (or its orthologue), which dissociates ubiquitinated LD proteins from LDs, such as oleosin or ATGL, thus affecting the rate of lipolysis [187,188,189].

### 5.3. Diatom-Specific LD Proteins

So far, diatom LD proteins have only been isolated from weakly silicified marine raphid pennate species from the order *Naviculales*, *F. solaris* and *P. tricornutum* [37,38,39,147,190,191]. Although centrics and pennates are genetically quite disparate, similarities in the metabolic responses of *T. pseudonana* [89] and *P. tricornutum* [85] to N starvation suggests that we could expect some general similarities between the LD proteomes of the *Naviculales* and other diatoms. The first diatom LD proteome reported was from *F. solaris* [190]. LD formation was induced by nutrient starvation, cells were then fractured by bead-beating and isolated by centrifugation. Proteins from the LD fraction were then precipitated, run on SDS-PAGE, digested in gel with trypsin and sequenced by LC/MS. This procedure identified 41 proteins, almost half of which were also identified in the aqueous phase of their centrifugation. Of the proteins only present in the LD fraction, HECTAR predicted that some of the proteins were targeted to the chloroplast, mitochondria and ER. Although such predictions might not definitely rule-out those proteins as being LD proteins, they may nevertheless be evidence of contamination from other compartments. Of the proteins predicted to target to the cytosol, 5 were predicted to contain transmembrane domains and two were confirmed to localize to LDs in vivo by expression of GFP-fusion constructs. One of them, g6574/G16118 co-localized to the ER and was predicted to contain a potassium channel. The other, later named Diatom Oleosome-Associated Protein 1 (DOAP1), was shown to possess a cleavable ER-targeting signal peptide, a proline-rich hydrophobic C-terminal domain and a quinoprotein alcohol dehydrogenase-like domain [151]. Interestingly, quinoprotein alcohol dehydrogenase-like domains are also present on a component of the ER-membrane protein complex in opsithokonts, EMC1 [192]. EMC1 is capable of tethering the ER to the mitochondrion, to facilitate the exchange of phospholipids [193]. It is possible DOAP1 performs a similar function for diatom LDs.

Since the seminal work of Nojima et al. (2013), later attempts to sequence diatom LD proteins yielded 5, 86, and 32 total proteins, using variations of generally similar isolation methods [37,38,40]. *P. tricornutum* has a homologue of DOAP1, named PtLDP1, which contains a WD40/YVTN repeat domain and a proline-rich hydrophobic C-terminal domain [191]. Although WD40 repeats are a common motif, they are found in proteins which regulate and localize to LDs in animals [194,195,196]. PtLDP1 was identified by MS of isolated LDs and its in vivo localization on LDs was confirmed with an EYFP-fusion construct [191]. Over-expression of PtLDP1 resulted in the upregulation of genes encoding the TAG biosynthesis enzymes DGAT2 and GPAT and the fatty acid biosynthesis enzymes FABI and FABG, increased TAG content, total lipid content and LD size [191]. Knock-down of PtLDP1 by RNA silencing had the inverse effects.

Another LD protein, StLDP was identified by LC/MS of LDs isolated from *P. tricornutum* [37,38,147] and later in vivo localization to LDs was confirmed with an EGFP-fusion construct [197]. StLDP expression was shown to correlate with LD surface area [37]. Although over-expression of StLDP did not induce LD formation, it did result in higher neutral lipid accumulation during N starvation, as well as the accumulation of larger and more LDs per cell compared to WT [197]. The operational mechanism of StLDP remains unclear, particularly since it bears no homology to LD proteins from the *Viridiplantae* or *Opisthokonta*. However, Yoneda et al. (2016) [37] did identify a hydrophobic motif conserved among other stramenopiles that was enriched with prolines and predicted to form transmembrane helices. The abundance and consistency of StLDP identification on LDs indicates that it would be a good candidate as an organelle marker protein, at least for LDs formed during N starvation.

### 5.4. Protein Chaperones, Storage and Degradation

The heat shock protein, Hsp70, has also been identified in multiple diatom LD proteomic studies [37,38,39,147] and has been verified to localize to LDs in other unrelated organisms [198]. In HepG2 cells, over-expression of Hsp70 resulted in increased accumulation of LDs and upregulation of lipogenic enzymes, while knock-down had the opposite effect [199]. In the *Naviculales* however, suppression of Hsp70 by the inhibitor VER-155008 resulted in larger LDs during both TAG synthesis and lipolysis [39]. Such evidence suggests that the operational mechanism of Hsp70 extends beyond nebulously functioning as a chaperone. In eukaryotes, transmembrane alpha-helices are typically inserted into membranes during post-translation or co-translational translocation, involving the Sec61 translocon, ribosomes and BiP [200,201,202]. BiP is a member of the Hsp70 protein family and has also been identified in LD proteomic studies in diatoms [38,147] and other organisms [17,203,204]. The plant LD protein oleosin has been shown to require the Sec61 translocon for insertion to the ER membrane prior to LD translocation in yeast [205]. The trans-kingdom demonstration of this targeting mechanism by Beaudoin et al. [26] suggests that it may also be conserved and therefore operating in diatoms. Such a mechanism would be consistent with the close associations observed between the ER and LDs and may also help explain why ribosomal proteins are also consistently identified in LD proteomics experiments.

Lupette et al. [38] suggest BiP may be functioning as part of the ER-associated degradation (ERAD) pathway, which would also be consistent with the identification of several other ERAD and proteasomal proteins in the LD proteome. Indeed, ERAD has been shown to be a conserved mechanism that regulates LD proteins in other eukaryotes [206]. During nitrogen starvation, diatoms undergo large-scale degradation of proteins [85]. Partially unfolded proteins can aggregate, causing cytotoxicity [207] and disruption of proteasomal degradation [208]. ERAD directs misfolded proteins to the proteasome [209]. It has been previously proposed that LDs function as an “escape hatch” for misfolded proteins to exit the ER during ERAD [210] and could perhaps serve as a physical highway to the proteasome for membrane-embedded ERAD proteins that are insoluble in the cytosol. In other eukaryotes, LDs co-ordinate their functions with both proteasomal and autophagic activity and can serve as a convergence site for both [211,212,213,214,215]. This functions to prevent the formation of cytotoxic protein aggregates and mitigates ER stress [216,217,218]. In diatoms, specialized machinery derived from ERAD, termed symbiont-specific ERAD-like machinery (SELMA), facilitates protein translocation across the periplastidial membrane [219]. Interestingly, SELMA involves ubiquitination and the APTase Cdc48, which, as mentioned previously, regulate LD lipolysis in other eukaryotes. The relationship between diatom LDs and ERAD may be complex and warrants further investigation.

LDs may also sequester functional proteins. For example, too few, too many or free histones can have various cytotoxic effects, such as increasing DNA damage sensitivity [220,221]. In *Drosophila* embryos, LDs sequester functional histones to modulate histone content during critical stages of development [222]. LD-sequestered histones also form an anti-bacterial defense system [223]. Histones have also been identified in diatom LD proteomics experiments [38,147], although whether they have a similar function to those in *Drosophila* is yet to be determined. The temporary storage of functional proteins could also help explain the presence of many unexpected proteins in diatom LD proteomics experiments, such as components of the photosynthetic apparatus. This could perhaps facilitate faster recovery from stress conditions by more rapid reassembly of photosynthetic machinery.

Since LDs may sequester proteins for a variety of purposes not directly related to lipid metabolism and LDs may be functionally connected to multiple compartments, robust experimentation will be required to fully characterize the precise functions, regulatory mechanisms and spatiotemporal partitioning of all LD proteins. It could take decades of work to gain a comprehensive understanding of a single model species, let alone the entire *Bacillariophyceae*. The progress in recent years warrants enthusiasm but is only the beginning of a long and tedious road of discovery.

## 6. LD Biotechnology

Diatoms have gained interest as photosynthetic sources of nutraceutical lipids, biofuels, isoprenoids and aquacultural feed [224,225,226]. The development of industrial scale microalgal cultivation has been constrained by low yields not yet competitive with alternative sources, such as fossil fuels, chemical synthesis, plants or heterotrophic organisms. However, diatoms and other algae have the potential benefit of simultaneously sequestering CO_2_, not taking up arable land and the ability to be cultivated with saline or waste water [227,228,229]. In this context, numerous metabolic engineering efforts have been undertaken to enhance TAG accumulation in diatoms. Such studies can also provide novel insights into fundamental aspects of diatom lipid metabolism.

A classical problem of metabolic engineering has been the avoidance of pleiotropic effects. Metabolic pathways are immensely complex systems that involve overlapping, competing, branched and redundant regulatory mechanisms which have evolved by natural selection over the course of hundreds of millions of years [230]. Early attempts at metabolic engineering either modulated singular key enzymatic steps within a pathway or the first committed step in the target pathway to sequester necessary precursors [231]. Unfortunately, such strategies tend to result in pleiotropic effects, such as genetic co-suppression, depletion of precursor pools, allosteric regulation or other undesired biochemical and phenotypic effects [231]. To solve these issues, ideally one would be able to sequester a desired metabolite in a protective pocket that would shield the rest of the cell from its accumulation. That is in fact the very function that LDs have evolved to perform.

In plants, use of sink organs to sequester metabolites, such as fruit, has been proposed as a promising strategy to avoid pleiotropic effects while enhancing isoprenoid content [232]. It is also possible to modify specific organelles to create a metabolic sink at an intracellular level. For example, increasing the size and number of plastids in tomato fruit resulted in an increase in isoprenoid content [232,233]. In an effort to increase TAG content in leafy plant tissues, a metabolic engineering strategy has been devised, termed “push, pull, package and protect” [234,235]. “Push” referring to the upregulation or over-expression of upstream reactions, such as fatty acid synthesis, “pull” referring to increasing the expression of the TAG synthesis pathway, “package” referring to the sequestration of TAG within LDs by expressing proteins involved in LD biogenesis, lipid trafficking to LDs or maintaining LD structural stability. Finally, “protect” the accumulated TAG by blocking access to lipases or suppression of downstream catabolic reactions, such as lipolysis or beta-oxidation.

Recently, LD formation was induced in *Nicotiana* plastids in an attempt to sequester terpenoids [236]. However, plastids are components of central metabolism and as such, enhancement or modulation risks affecting cell growth rate, mortality and so forth. Furthermore, any LDs accumulated within plastids will be constrained by the size of the plastid. Cytosolic LDs offer the benefit of less direct impact on central metabolism and having greater room for expansion. In *Nicotiana*, terpenoids were successfully sequestered to cytosolic LDs simply by over-expressing genes responsible for LD biogenesis and sesqueterpene synthesis [237,238]. Furthermore, fusion of terpenoid synthesis enzymes with the *Nannochloropsis* LD protein NoLDSP, resulted in the localization of terpenoid synthesis to LDs and sequestration therein [239]. Unlike in plant leaf tissues, metabolic engineering of diatom TAG accumulation benefits from the existence of LD biogenesis and regulatory systems already in place. Various steps within the “push, pull, package, protect” paradigm have already has been accomplished in diatoms by over-expression of enzymes implicated in fatty acid and TAG biosynthesis [114,117,240,241,242], modulating the acyl-CoA pool [243] or repression of lipid catabolism [124,125,244]. A deeper understanding of the mechanisms by which proteins are targeted to LDs could facilitate the engineering of LD-localized enzymes or metabolic pathways, which could allow for the customization of LD contents.

LDs may not be the ultimate solution to the challenges facing diatom cultivation. The main disadvantage is of course that the lipids still exist within the cell itself, which not only requires more investment in harvesting technique but also requires killing the algae to get at the product inside. The alternative to this is what has been called “milking,” that is, the extracellular secretion of lipids [245,246]. This could be accomplished either by the secretion of free lipids or extracellular vesicles. Genetic engineering is also not necessarily the ultimate answer to overcoming the challenges of diatom biotechnology and the key may lie in identifying an ideal species for cultivation by bioprospecting. Although we do not know the absolute number of extant diatom species, estimates range from tens of thousands to one or two hundred thousand species [247,248]. Given such a wealth of species diversity, it can be assumed that suitable oleaginous species are out there, waiting to be discovered.

## 7. Conclusions

LDs operate at the nexus of multiple metabolic pathways and intracellular systems, as well as at the nexus of multiple sub-disciplines, including biochemistry, ecophysiology, and evolution. Recent decades have seen a massive increase in research about both lipid droplets and diatoms. Synthesis between these two niche fields is in a nascent stage, but holds immense promise for myriad biotechnological applications. Yet, there remain many outstanding questions that beckon to be answered. What are the precise functions and relationships of LDs, and their associated proteins? How many proteins are directly involved in LD functions? Are proteins identified in isolated LDs representative of active functional proteins, stored functional proteins, stored proteins en route to degradation pathways, evidence of inter-organelle membrane bridges, or simply contaminants? What are the specific steps in LD biogenesis, turnover and degradation in diatoms? What are the differences between different strains, species, and genera? Can LD protein targeting mechanisms be manipulated to empower metabolic engineering? It is our hope that this review will be helpful to the next generation of scientists seeking to understand this curious organelle, serving as a roadmap towards past and future advances.
